# Endoscopic Submucosal Dissection of Gastric Neoplastic Lesions: An Italian, Multicenter Study

**DOI:** 10.3390/jcm9030737

**Published:** 2020-03-09

**Authors:** Raffaele Manta, Giuseppe Galloro, Francesco Pugliese, Stefano Angeletti, Angelo Caruso, Francesco P. Zito, Santi Mangiafico, Riccardo Marmo, Angelo Zullo, Gianluca Esposito, Bruno Annibale, Massimiliano Mutignani, Rita Conigliaro

**Affiliations:** 1 Gastroenterology and Digestive Endoscopy, General Hospital, 06129 Perugia, Italy; 2 Surgical Digestive Endoscopy, Department of Clinical Medicine and Surgery, Federico II University, 80055 Naples, Italy; giuseppe.galloro@unina.it; 3 Digestive Endoscopy Unit, Niguarda Hospital, 20162 Milan, Italy; francesco.pugliese@ospedaleniguarda.it (F.P.); massimiliano.mutignani@ospedaleniguarda.it (M.M.); 4 Department of Clinical Sciences and Translational Medicine, Sant’Andrea Hospital, Sapienza University. 00189 Rome, Italy; stefanoangeletti63@gmail.com (S.A.); gle.esposito@gmail.com (G.E.); bruno.annibale@uniroma1.it (B.A.); 5 Digestive Endoscopy Unit, S. Agostino-Estense Hospital, 41126 Modena, Italy; an.caruso@ausl.mo.it (A.C.); francescopaolo.zito@unina.it (F.P.Z.); sa.mangiafico@ausl.mo.it (S.M.); r.conigliaro@ausl.mo.it (R.C.); 6 Gastroenterology and Digestive Endoscopy, L. Curto Hospital, Polla, 84036 Salerno, Italy; riccardo.marmo@gmail.com; 7 Gastroenterology and Digestive Endoscopy, Nuovo Regina Margherita Hospital, 00153 Rome, Italy; angelozullo66@yahoo.it

**Keywords:** endoscopic submucosal dissection, early gastric cancer, precancerous lesions, stomach

## Abstract

Endoscopic submucosal dissection (ESD) allows removing neoplastic lesions on gastric mucosa, including early gastric cancer (EGC) and dysplasia. Data on ESD from Western countries are still scanty. We report results of ESD procedures performed in Italy. Data of consecutive patients who underwent ESD for gastric neoplastic removal were analyzed. The *en bloc* resection rate and the R0 resection rates for all neoplastic lesions were calculated, as well as the curative rate (i.e., no need for surgical treatment) for EGC. The incidence of complications, the one-month mortality, and the recurrence rate at one-year follow-up were computed. A total of 296 patients with 299 gastric lesions (80 EGC) were treated. The *en bloc* resection was successful for 292 (97.6%) and the R0 was achieved in 266 (89%) out of all lesions. In the EGC group, the ESD was eventually curative in 72.5% (58/80) following procedure. A complication occurred in 30 (10.1%) patients. Endoscopic treatment was successful in all 3 perforations, whereas it failed in 2 out of 27 bleeding patients who were treated with radiological embolization (1 case) or surgery (1 case). No procedure-related deaths at one-month follow-up were observed. Lesion recurrence occurred in 16 (6.2%) patients (6 EGC and 10 dysplasia). In conclusion, the rate of both *en bloc* and R0 gastric lesions removal was very high in Italy. However, the curative rate for EGC needs to be improved. Complications were acceptably low and amenable at endoscopy.

## 1. Introduction

The possibility of removing early neoplastic lesions on gastric mucosa by endoscopic submucosal dissection (ESD) is a definite advantage for both patients and health resources utilization [1]. This procedure allows treatment of early gastric cancer (EGC) and non-invasive neoplasia—i.e., low- and high-grade dysplasia. In detail, ESD consists of performing *en bloc*, margin-negative (R0) resection, and curative resection, thus avoiding surgery and sparing the stomach in those patients suitable for the procedure [2]. First introduced in Japan twenty years ago, the use of the ESD technique, particularly in removing gastric lesions, is still limited in Western countries [3]. Likewise, this depends on both a lower incidence of gastric cancer and its detection in an advanced stage in Western countries, where screening programs are lacking [4]. Indeed, the detection rate of early gastric cancer (EGC) still remains low in Western endoscopic units. However, in the last decade, the ESD procedures have been increasingly performed in non-Asian countries, mainly in tertiary centers. This multicenter study aimed to evaluate the results of the ESD procedure for gastric lesion removal in Italy, in terms of technical success, clinical outcome, and complications.

## 2. Materials and Methods

### 2.1. Patients and Procedures

This was a retrospective analysis of prospectively collected series of patients diagnosed with gastric superficial lesions who underwent ESD between January 2009 and December 2019. In each participating center, endoscopic, histological, and clinical data of consecutive patients who underwent gastric ESD were gathered in an electronic database, which were anonymously analyzed as aggregate data for the center. For the endoscopic description of lesions, the Paris classification was used [5], while histological examinations were performed accordingly to the Vienna classification [6].

For all lesions (EGC and dysplasia), the technical success was calculated based on the *en bloc* removal of tissue, and the R0 resection when free lateral and vertical margins were found at histology. Free margin was defined as at least 2 mm distance from the lesion to the margin in the resected specimen. The curative rate for EGC was defined according to Japanese and European criteria, and corresponded to no need for surgical treatment (i.e., R0 removal and absence of histological characteristics at risk of metastasis) [6,7]. All operators who performed the ESD procedures had at least 10 years’ experience in therapeutic endoscopy, and previously performed specific training in Japan. The ESD was performed under deep sedation with propofol, by following the standard procedure.

The rate of complications, including early (<24 h) and delayed (≥24 h), was calculated, as well as the one-month mortality rate. Recurrence was defined as detection of neoplastic tissue at the scar during 12 months of follow-up.

All patients were informed about advantages and disadvantages of endoscopic procedure, as well as on potential complications, and signed informed consent for the procedure and to anonymously use data for scientific purpose. Since no experimental drugs were administered, no additional costs or procedures for the patients were required, no identification of patients was allowed, and no funds were received, our Investigational Review Board waived formal review and approval, deeming the study to be an extension of existing clinical practice.

### 2.2. Statistics

The frequency and percentages with their 95% confidence interval (CI) were calculated for each observation. The curative rate for EGC was calculated at both intention-to-treat (ITT) (all patients in whom ESD removal was attempted) and per protocol (PP) (patients with R0 removal) analysis. The chi-squared test or Fisher’s exact test, as appropriate, was used for comparing categorical variables among groups, as well as before pooling the estimates to investigate the heterogeneity among centers.

## 3. Results

Data from five centers (Perugia, Modena, Naples, Milan, Rome) were collected, and a total of 296 patients (53% males; mean age: 69 years, range 42–88 years) with 299 superficial lesions were treated. The lesions were localized in distal stomach (antrum/angulus) in 200 cases and in the proximal stomach in 99 cases. The mean diameter of the removed lesion was 24.5 mm, ranging from 10 to 70 mm. The median time of procedure was 76 min (range: 18–187). The endoscopic and histological characteristics of removed lesions are provided in Table 1.

No statistically significant difference in main outcomes emerged among centers, so that data were pooled. The *en bloc* resection was successful in 292 (97.7%; 95% CI = 95.2–98.9) out of 299 lesions. The R0 was achieved in 266 (91.1%, 95% CI = 87.3–93.8) out of 292 properly removed lesions, the rate being statistically significant (*p* < 0.001) and lower for EGC (64/80, 80%, 95% CI = 70–87.3) than other lesions (202/212, 95.3%, 95% CI = 91.5–97.4). In detail, in the EGC group, the ESD eventually failed to achieve R0 in 16 cases and to be therapeutic (histological risk factors present) in another 6 patients, accounting for a 72.5 % (58/80, 95% CI = 63–82.3) and 90.6% (58/64, 95% CI = 83.5–97.8) curative rate at ITT and PP analyses, respectively. A total of 15 (75%, 95% CI = 53–89) out of 22 EGC patients without curative ESD underwent surgical treatment, 5 were judged unfit for surgery and were retreated at endoscopy (ongoing follow-up). The remaining 2 patients refused surgical intervention despite an exhaustive explanation. Both these patients, one with positive lateral margin and one with positive vertical margin, were free of recurrence at 12 and 15 months, respectively.

**Figure 1:**
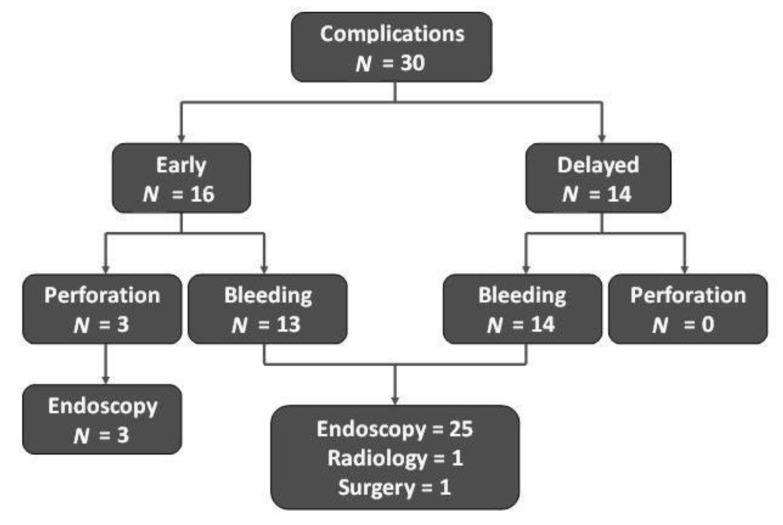
Post-procedural complications and their management.

Overall, a complication occurred in 30 (10.1%) patients, and their management is summarized in the Figure 1. No procedure-related deaths at one-month follow-up were observed. Within 12 months of follow-up, a lesion recurrence was detected in a total of 16 (6.2%, 95% CI = 3.2–9) out of 260 patients following a curative ESD procedure. In detail, 9 lesions (3 EGC and 6 dysplasia) recurred within 3 months, and other 7 lesions (3 EGC and 4 dysplasia) between 3 and 12 months, accounting for a cumulative recurrence rate of 10.3% (6/58; 95% CI = 2.5–18.2) for EGC and 4.9% (10/200; 95% CI = 1.9–8.1) for dysplasia at one year. Patients with recurrent EGC underwent surgical treatment, while all dysplastic lesions were removed successfully at endoscopy.

## 4. Discussion

The mortality for gastric cancer remains high worldwide [8]. Particularly, in Western countries, this depends on the fact that diagnosis is generally performed at an advanced stage, when survival is dismal. However, by applying an appropriate sampling of gastric mucosa at endoscopy to allow gastritis staging at histology, it is possible to identify those patients at increased risk of gastric cancer who deserve a scheduled follow-up [9]. Moreover, by using virtual chromoendoscopy techniques and echoendoscopy, it is possible to identify and characterize both non-invasive neoplasia and EGC on gastric mucosa, susceptible of removal with ESD [10]. This therapeutic approach is largely used in Asia, and it has been increasingly performed in Western countries, including some Italian centers [11].

In the present study, we collected data from five centers for nearly 300 patients, including 80 EGCs, treated with ESD. Our data found a very high *en bloc* (97.6%) resection rate for all neoplastic lesions. This value is highly comparable to data from a recent meta-analysis on Asian series, showing an *en bloc* resection rate of 93.4% for 2387 cases [1]. Similarly, we found an overall high (91.1%) R0 resection rate. However, in our series, the R0 rate for EGC (80%) was distinctly lower than that of other lesions, and the curative rate was 72.5%. These results seem to be in line with the curative rates reported in other European countries, where values ranged widely from only 7.7% to 91.7% (Table 2) [12,13,14,15,16,17]. Specifically, a learning curve could play a role in the overall results of our series over a long period, due to expected worst outcomes in the initial experience. Indeed, data of gastric ESD procedures performed in Italy in 2016 and 2017 in dedicated centers showed a curative rate of 86.7% and 82%, respectively [11]. Cumulatively, data from Western countries appear to be lower than the 91.7% for 2017 reported in Asian series [1]. Moreover, in our study, a local recurrence of EGC was observed in 10% of patients at one-year follow-up, a value distinctly higher than in Asian studies where it was only 2.3% for 1460 cases [1]. Therefore, an improvement is needed in Western countries with respect to correctly staging the EGC before procedure, removing it at endoscopy, and properly assessing the removed tissue at histology. Indeed, recurrence within 3–12 months most likely represents an incorrect staging of lesion at histology rather than a *de novo* development of cancer. On the other hand, a recent long-term study found no residual disease after gastrectomy in as many as 75% of patients in whom ESD was judged to be non-curative [18].

The overall complication rate (10.1%) was acceptably low, and the complications mainly consisted in bleeding that was successfully managed at endoscopy in the majority of cases, as well as the few perforations. Of note, our results were consistent with the overall 10.4% reported in Asian studies (7.2% bleeding for 1989 cases and 3.2% perforation for 2672 patients) [1]. Moreover, no procedure-related death at one-month follow-up was observed. Therefore, the ESD procedure may be considered safe and advantageous as compared to surgery [1].

## 5. Conclusions

In conclusion, this is a large Italian series on ESD procedures for neoplastic lesion removal on gastric mucosa. Both *en bloc* and R0 resection rates were very high, with acceptably low incidence of complications, whereas the curative rate for EGC needs to be improved to achieve the results reported in Asia.

## Figures and Tables

**Table 1:** Characteristics of lesions at endoscopic and histology.

Findings	*N* = 299 Lesions
Endoscopic features *	
0–1 s	20
IIa	110
IIb	42
IIc	9
IIa + IIb	18
Is-IIa	2
**Histological features**	
EGC	80
High-grade dysplasia	114
Low-grade dysplasia	103
NET	2

* According to Paris classification. EGC: Early gastric cancer. NET: Neuroendocrine tumor.

**Table 2:** Curative rates for early gastric cancer following endoscopic submucosal dissection (ESD) reported in some European countries.

Reference	Country	EGC (*N*)	Curative Rate (%)
Kim et al. [13]	UK	13	7.7
Probst et al. [14]	Germany	122	63.9
Petruzziello et al. [12]	Italy	44	65.9
Karpińska-Kaczmarczyk et al. [15]	Poland	41	70.7
Mocker et al. [16]	Germany	19	73.1
Maselli et al. [12]	Italy	502	81.7
Catalano et al. [17]	Italy	12	91.7
